# Development of a performance-derived nutritional value for a microencapsulated gut health additive from a meta-analysis of 35 trials and its *in vivo* validation in broilers

**DOI:** 10.3389/fvets.2026.1888365

**Published:** 2026-10-09

**Authors:** Giulia Giovagnoni, Andrea Bonetti, Mauro Di Benedetto, Benedetta Tugnoli, Andrea Piva, Ester Grilli

**Affiliations:** 1 Vetagro SpA, Reggio Emilia, Italy; 2 Independent Researcher, Santarcangelo di Romagna, Italy

**Keywords:** feed additive, gut health, meta-analysis, microencapsulation, nutrient absorption, nutritional value, poultry nutrition

## Abstract

Feed additives targeting gut health are widely used to improve efficiency in broilers, yet their nutritional value is rarely quantified, especially when containing ingredients like botanicals and organic acids. The objective of the study was to develop a nutritional value for a microencapsulated blend of botanicals and organic acids (mBOA; AviPlus^®^P) and validate the approach *in vivo*. A meta-analysis of 35 trials (2009–2022) was carried out comparing mBOA at 200–500 mg/kg vs. control groups, both under non-medicated cereal-soy diets for durations spanning 34–48 days. Random-effects (REML) models summarized effects on final body weight (FBW) and standardized feed conversion (FCRst). Across studies, mBOA increased FBW (+38 g; *P* = 0.009) and improved FCRst (−0.04 points; *P* = 0.001). The observed performance gains were back-converted into dietary energy and protein equivalences to parameterize a performance-derived nutritional value for mBOA at 300 mg/kg, resulting in 60.58 kcal of Metabolizable Energy (ME) and 3.88 g of crude protein (CP) per kg of diet. The nutritional value was then tested in 810 Ross 308 males (15 pens/treatment) allocated into three groups: (1) a group fed with a standard diet (STD), (2) a group fed with a reformulated diet (REF = −80 kcal ME/kg and −2% CP and digestible amino acids), and (3) a group fed with the same reformulated diet and supplemented with mBOA at 300 mg/kg (APP). Overall (d 0–41), REF showed higher average daily feed intake (ADFI) and worse feed conversion ratio (FCR) than STD (1.56 vs. 1.53), while APP was intermediate (1.54) and numerically closer to STD (*P* = 0.003). APP improved CP digestibility compared to STD (*P* = 0.038) and fat digestibility to REF (*P* < 0.001), partially restoring AMEn (APP 2852 vs. REF 2827 vs. STD 2873 kcal/kg DM; *P* = 0.003). The findings obtained using this multi-step approach support the application of mBOA to partially offset reduced-density formulations without compromising efficiency, providing formulation-ready values for broiler diets.

## 1 Introduction

In the current socio-economical context, feed represents one of the highest costs (over 60%) in poultry production (1), with the main objective of formulations being to minimize costs while maintaining performance (2). Feed reformulation refers to the process of adjusting the composition and nutrient density of poultry diets while maintaining nutrient balance. This practice can be implemented using additives that target feed efficiency and for which a nutritional value is established. Traditionally, exogenous enzymes have been a key category of additives for feed reformulation and optimization, as they increase nutrient digestibility (3). However, more recently other categories of feed additives have been widely evaluated as tools for feed optimization purposes, thanks to their gut health mechanisms of action. Among these, ingredients like botanicals and organic acids have been broadly studied and their employment in monogastric animals relies on their recognized anti-inflammatory, antioxidant, and antibacterial properties (4, 5). One of the main challenges in the use of these compounds is ensuring their delivery to the intestine, where their biological functions are required: in fact, without adequate protection, most of them are degraded or absorbed in the stomach and fail to reach the intestine (5–7). Lipid microencapsulation of feed additives allows the active ingredients to by-pass the gastric environment, thus being slowly released throughout the intestine of the animal, where they are needed for gut health purposes (8).

Homeostasis is fundamental for gut health: disruption to this balance may be triggered by many causes, such as feed antigens, dysbacteriosis, and heat/cold stress, and have adverse consequences on animal growth performance (9). In fact, intestinal inflammation imposes a measurable energetic burden: increased permeability and dysbiosis trigger immune activation, which diverts energy and amino acids from growth toward immune activation and re-establishment of tissue homeostasis. Previous research indicates that immune activation has a cost that reallocates nutrients available for performance purposes, thus reducing growth and impairing feed conversion ratio (FCR) (10–12). These mechanisms emphasize that controlling intestinal inflammation is not only profitable under physiological but also nutritional point of view, because it improves the efficiency with which dietary energy and nutrients are effectively converted into animal growth.

In this study, the nutritional value of a microencapsulated feed additive based on botanicals and organic acids (mBOA) was evaluated. Thanks to the synergy of ingredients, the multifaceted mechanism of action of mBOA leans on the beneficial regulation of the microflora, the improvement of the host intestinal barrier, the modulation of the immune system, and on the increased nutrient digestibility and absorption as the latest findings (13–16). Altogether, these modes of action concur to produce a positive effect on gut health that translates into enhanced animal growth performance and feed efficiency (13). Current literature on gut health feed additives predominantly reports their effects on growth performance, yet rarely progresses toward investigating their nutritional role and formulation-ready values. Establishing such nutritional values requires the availability of a robust database of performance data that should be consolidated meta-analytically and supported by *in vivo* validations of the outcomes. Starting from the peer-reviewed evidences on the mBOA mechanisms of action, the aim of the study was the development of a performance-derived nutritional value for mBOA, based on a robust meta-analysis and validated by an *in vivo* trial in broilers.

## 2 Materials and methods

### 2.1 Meta-analysis database

#### 2.1.1 Study eligibility criteria

A total of 41 trials evaluating the effect of mBOA (AviPlus^®^P, Vetagro S.p.A., Reggio Emilia, Italy; EU feed additive N. 4d3; Protected by European Patent EP2185005 B2, US Patent US8722740 B2, Canadian Patent CA2932088C, and corresponding granted patents in multiple jurisdictions worldwide) on broiler growth performance, were identified within the internal historical trial database. Only trials meeting the following criteria were included: (i) presence of a parallel control group, (ii) controlled conditions without exposure to any experimental or artificial challenge, and (iii) supplementation of mBOA on top of the basal diet in treated groups at inclusion levels ranging from 200 to 500 mg/kg, for the whole duration of the trial, up to a commercially relevant slaughter age (34–48 days). Considering the criteria, six trials were excluded: two for the presence of a controlled disease challenge (Necrotic Enteritis); one for a protocol deviation, since mBOA was not administered for the entire duration of the study; three for inconsistencies across recorded production parameters (Figure 1). The remaining 35 trials met all eligibility criteria and were included in the meta-analysis (Table 1).

**Figure 1:**
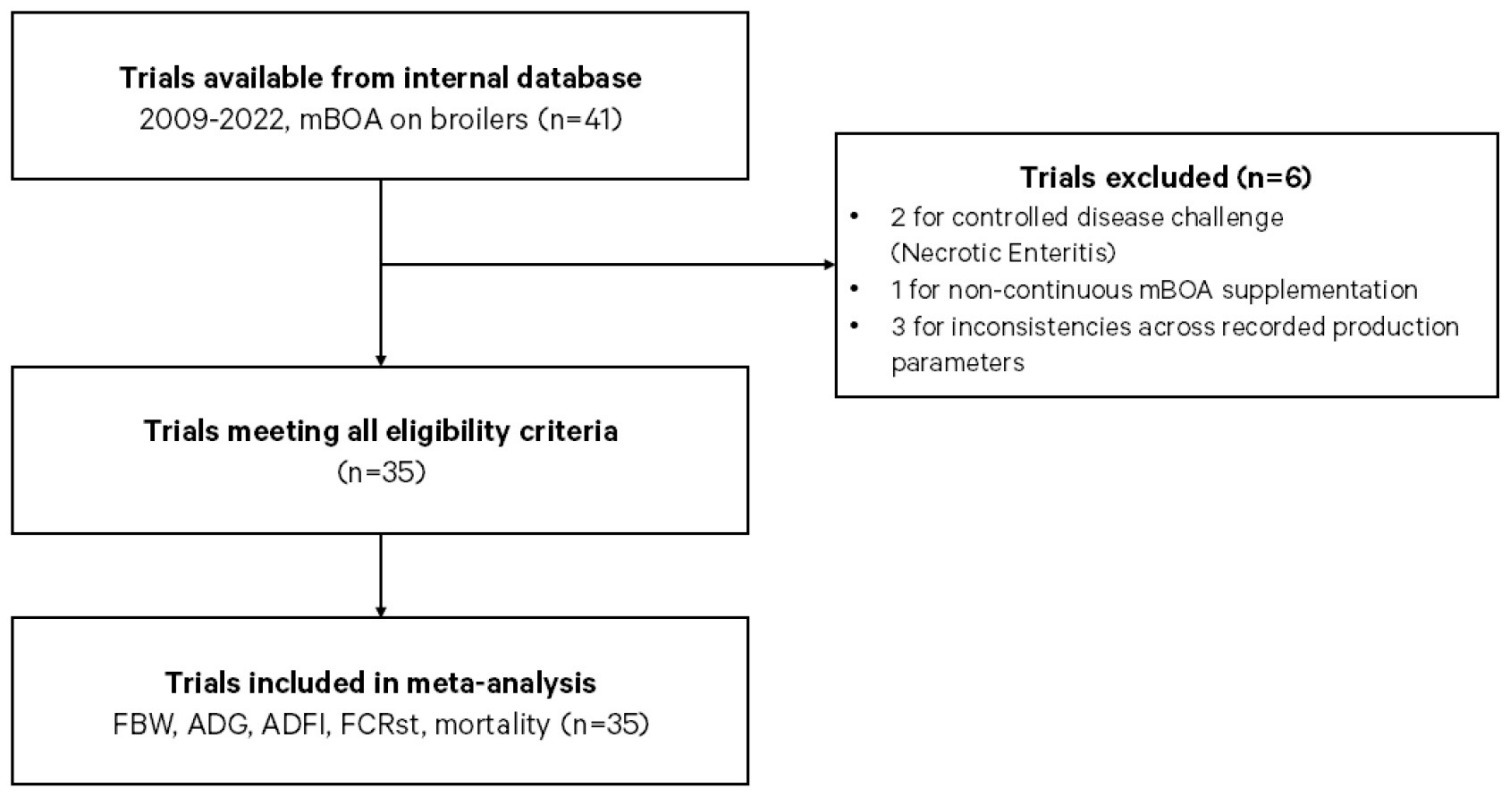
Flow diagram of trial identification, exclusion, and inclusion for the meta-analysis of mBOA effects on broiler growth performance.

**Table 1:** Overview of the trials included in the meta-analysis.

Study reference	Year of trial	Country	Genetics	Trial type	Trial duration, days	Control, heads	mBOA, heads	mBOA dosage, mg/kg
1	2011	Italy	Ross 308	Experimental	45	177	177	300
2	2011	USA	Cobb 500	Experimental	42	1,100	1,100	300
3	2012	USA	Cobb 500	Experimental	42	550	550	300
4	2014	Belgium	Ross 308	Experimental	42	450	450	300
5	2020	UK	Ross 308	Experimental	42	300	300	300
6	2021	Germany	Ross 308	Experimental	34	50	50	500
7	2013	Poland	Not specified	Field	43	40,000	20,000	300
8	2013	Poland	Not specified	Field	40	31,000	31,000	300
9	2013	Poland	Not specified	Field	38	2,000	400	300
10	2013	China	Ross 308	Field	41	12,000	12,000	250
11	2013	China	Ross 308	Field	41	31,000	31,000	250
12	2013	Slovak Republic	Cobb 500	Field	42	15,000	15,000	300
13	2013	Slovak Republic	Cobb 500	Field	42	15,000	15,000	300
14	2019	Ukraine	Cobb 500	Field	42	46,500	46,500	300
15	2019	USA	Ross 308	Experimental	42	120	120	500
16	2008	Italy	Ross 708	Experimental	38	360	360	200
17^a^	2009	Italy	Ross 708	Experimental	38	360	360	200
18^a^	2009	Italy	Ross 708	Experimental	38	360	360	200
19	2011	Italy	HUBBARD	Experimental	35	180	180	200
20	2011	Italy	HUBBARD	Experimental	35	180	180	500
21	2011	UK	Ross 308	Experimental	35	400	400	200
22	2011	UK	Ross 308	Experimental	35	400	400	500
23	2010	UK	Ross 308	Experimental	35	160	160	500
24	2018	France	Ross 308	Experimental	42	675	675	200
25	2018	France	Ross 308	Experimental	42	675	675	300
26	2018	France	Ross 308	Experimental	42	675	675	500
27	2022	Italy	Ross 308	Experimental	42	462	462	300
28	2013	Italy	Ross 308	Field	43	780	780	300
29	2013	Belgium	Ross 308	Experimental	42	340	340	300
30	2013	Slovakia	Ross 308	Field	42	13,000	13,000	300
31	2013	Slovakia	Cobb 500	Field	42	14,000	14,000	300
32	2014	Italy	Ross 308	Field	48	18,400	18,400	500
33	2012	USA	Cobb 500	Experimental	42	1,008	1,008	200
34	2012	Korea	Ross	Experimental	35	100	100	250
35	2012	Korea	Ross	Experimental	35	100	100	500

^a^Independent trials conducted in different periods of the same year.

Across such trials, diets were non-medicated and control groups received the standard diet (STD) without mBOA or any additional gut health feed additives. The selected studies were conducted between 2009 and 2022 in research centers or commercial farms across 11 countries, in either Europe, Asia, or United States, encompassing multiple broiler genetics. All diets were formulated using common cereal-soy bases, including wheat, corn, barley, and soybean meal.

#### 2.1.2 Data extraction and standardization

For each study, the following overall performance outcomes were extracted, when available, or calculated: average daily feed intake (ADFI), average daily gain (ADG), final body weight (FBW), and mortality. To allow comparison across trials, feed conversion ratio (FCR) values were standardized (FCRst) to account for differences in FWB. Specifically, FCR was adjusted by subtracting 0.01 units of FCR per additional 50 g of FBW, in accordance with industry technical recommendations for modern broilers.

#### 2.1.3 Statistical analysis

The meta-analysis was performed using IBM SPSS Statistics v30.0, employing the “Meta-Analysis Continuous” module. Treatment effects for continuous outcomes were expressed as unstandardized mean differences between mBOA-supplemented and control groups. Group means for each parameter were derived from pen/house-level production records. The standard error for each trial was calculated using the number of birds per group as the denominator. Pooled estimates were calculated using a random-effects model with inverse-variance weighting to account for between-study heterogeneity. The between-study variance (τ^2^) was estimated by restricted maximum likelihood (REML), and standard errors, confidence intervals, and 95% prediction intervals were adjusted using the truncated Knapp–Hartung method to provide robust inference. Subgroup analyses (experimental vs. field trials) were performed using the same random-effects model, with between-subgroup differences tested via the *Q*-between statistic. Meta-regression on mBOA dose (200–500 mg/kg) was performed using the same model, also jointly with Egger's regression-based test to assess Funnel plot asymmetry for each outcome. A leave-one-out sensitivity analysis, iteratively excluding one trial at a time and re-estimating the pooled effect, was conducted for FBW to assess the influence of individual trials on the pooled estimate. Significance was set at *P* ≤ 0.05.

### 2.2 Development and calculation of the performance-derived nutritional value

In order to determine the metabolizable energy (ME) and crude protein (CP) nutritional value of mBOA, an average dietary ME level of 3,000 kcal/kg of feed and CP content of 19% (together with the corresponding digestible amino acid levels) were considered according to the 2022 Aviagen Ross 308 Nutrition Specifications. This approach was employed in order to minimize the heterogeneity of the diets included in the meta-analysis.

Based on FCRst obtained from both control and mBOA groups, the ME (ME[feed], Equation 1) and CP (CP[feed], Equation 2) values recovered in the diet from each trial were calculated:


$$ME{\left[feed\right]}_{\frac{kcal\;ME}{kg\;feed}}=\frac{FCRst\left[control\right]-FCRst[mBOA]}{FCRst[mBOA]}\times 3,000\;$$ (1)

$$CP{\left[feed\right]}_{\frac{g\;CP}{kg\;feed}}=\frac{FCRst\left[control\right]-FCRst[mBOA]}{FCRst[mBOA]}\times 190\;$$ (2)

Afterwards, the ME and CP nutritional values for mBOA (ME[mBOA], Equation 3, and CP[mBOA], Equation 4) were determined taking into consideration the inclusion of mBOA (milligrams of mBOA per kg of feed) as follows:


$$ME{\left[mBOA\right]}_{\frac{kcal\;ME}{kg\;mBOA}}=\frac{ME[feed]\;\times 1,000}{Inclusion[mBOA]\times 1,000}$$ (3)

$$CP{\left[mBOA\right]}_{\frac{\;g\;CP}{kg\;mBOA}\;}=\frac{CP[feed]\;\times 1,000}{Inclusion[mBOA]\times 1,000}$$ (4)

The respective values for digestible amino acids (lysine, methionine + cysteine, methionine, threonine, valine, isoleucine, arginine, tryptophan, and leucine) were calculated as a percentage of the CP[mBOA] value. All the aforementioned conversions assume a proportional relationship between FCRst improvement and dietary ME/CP recovery.

Finally, the nutritional value for mBOA at 300 mg/kg was determined.

#### *2.3 In vivo* validation trial

#### 2.3.1 Experimental design

The *in vivo* trial was carried out at TRANSfarm, the test facility of KU Leuven, located in Lovenjoel, Bierbeek, The Netherlands. A total of 810 Ross 308 day-old male chicks were placed in 45 floor pens (18 birds/pen, pen size 1.3 m^2^) containing pine shavings and provided *ad libitum* access to feed and water throughout the study. Chicks were allocated into three different groups (*n* = 15 pens/group): (1) a group fed with a standard diet (STD) formulated according to Aviagen nutrition guidelines for Ross 308; (2) a group fed with a reformulated diet (REF); (3) a group fed with the same reformulated diet and supplemented with mBOA at 300 mg/kg (APP). The REF diet consisted of a low-density diet with a reduction of −80 kcal of ME and a −2% of CP and digestible amino acids, compared to the standard diet. The 2% reduction in CP is relative to a standard 19% CP diet, corresponding to approximately −0.38 absolute percentage points of CP. The composition of the diets for 4 different phases (Starter, Grower, Finisher I, and Finisher II) is reported in Table 2. Proximate analysis of the feeds was conducted to verify the actual compositional differences between the standard and reformulated diets.

**Table 2:** Diet composition of the experimental diets used in the validation trial.

	Starter (0–14 days)			Grower (14–28 days)			Finisher I (28–32 days)			Finisher II (32–41 days)		
Ingredients	STD	REF	APP	STD	REF	APP	STD	REF	APP	STD	REF	APP
Yellow corn	47.99	48.35	48.32	53.69	54.09	54.06	52.11	53.77	53.74	52.41	54.07	54.04
Soybean meal (47% CP)	33.10	31.39	31.39	27.11	25.14	25.14	22.88	21.37	21.37	22.88	21.37	21.37
Soft wheat	10.00	10.00	10.00	10.00	10.00	10.00	15.00	15.00	15.00	15.00	15.00	15.00
Soybean oil	2.04	1.00	1.00	2.02	1.00	1.00	2.31	1.00	1.00	2.31	1.00	1.00
Rapeseed meal (34% CP)	1.50	1.50	1.50	2.00	2.50	2.50	2.50	2.50	2.50	2.50	2.50	2.50
Sunflower meal (34% CP)	1.50	1.50	1.50	2.00	2.00	2.00	2.00	2.00	2.00	2.00	2.00	2.00
Limestone (calcium carbonate)	1.32	1.33	1.33	1.03	1.03	1.03	0.85	0.86	0.86	0.85	0.86	0.86
Monocalcium phosphate	0.65	0.64	0.64	0.50	0.49	0.49	0.33	0.33	0.33	0.33	0.33	0.33
Vitamin-mineral premix^a^	0.50	0.50	0.50	0.50	0.50	0.50	0.50	0.50	0.50	0.50	0.50	0.50
DL-methionine	0.38	0.37	0.37	0.31	0.29	0.29	0.30	0.29	0.29	0.30	0.29	0.29
L-lysine HCl	0.33	0.35	0.35	0.27	0.27	0.27	0.30	0.32	0.32	0.30	0.32	0.32
Sodium bicarbonate	0.23	0.24	0.24	0.20	0.21	0.21	0.23	0.24	0.24	0.23	0.24	0.24
Sodium chloride	0.19	0.19	0.19	0.21	0.21	0.21	0.20	0.19	0.19	0.20	0.19	0.19
L-threonine	0.17	0.17	0.17	0.12	0.11	0.11	0.13	0.13	0.13	0.13	0.13	0.13
L-valine	0.08	0.08	0.08	0.03	0.03	0.03	0.05	0.05	0.05	0.05	0.05	0.05
Phytase (5,000 FTU)	0.01	0.01	0.01	0.01	0.01	0.01	0.01	0.01	0.01	0.01	0.01	0.01
mBOA (AviPlus^®^P)	0.00	0.00	0.03	0.00	0.00	0.03	0.00	0.00	0.03	0.00	0.00	0.03
Wheat bran (15%−18%)	0.00	2.39	0.00	0.00	2.11	2.11	0.00	1.14	1.14	0.00	1.14	1.14
TiO_2_ (0.3%)	0.00	0.00	0.00	0.00	0.00	0.00	0.30	0.30	0.30	0.00	0.00	0.00

^a^Premix providing, per kg of diet: vitamin A, 10,000 IU; vitamin D3, 2,500 IU; vitamin E, 50 mg; vitamin K3, 2.3 mg; vitamin B1, 2.0 mg; vitamin B2, 7.5 mg; vitamin B6, 3.5 mg; vitamin B12, 20 μg; niacin, 35 mg; D-pantothenic acid, 12 mg; choline chloride, 460 mg; folic acid, 1.0 mg; biotin, 0.2 mg; iron, 80 mg; copper, 12 mg; manganese, 85 mg; zinc, 60 mg; Iodine, 1.2 mg; selenium, 0.25 mg.

The study lasted 41 days. The measurement of body weights (BW) and feed offered were planned on days 14, 28, and 41 to assess growth performance.

In order to assess nutrient digestibility, a TiO_2_ tracer was added (0.3%) as digestibility marker in the Finisher I diets (Table 2). On day 31 and 32, fecal samples were collected per pen during a 4–8 h sampling period by placement of tetra paper in each pen. Collected fecal samples were pooled to obtain a representative homogeneous sample (200–400 g) and stored at −20 °C. Fecal digestibility of dry matter (DM), crude protein (ISO 5983-2), crude fat (ISO 6492 B), crude ash (ISO 5984-2022), and gross energy (ISO 9831) were assessed. Apparent metabolizable energy corrected for nitrogen (AMEn, Equation 5) was calculated using the following formula:


$$\begin{array}{r} AMEn_{\frac{kcal}{kg}}=GEfeed_{\frac{kcal}{kg}}-(\frac{GE\;feces_{\frac{kcal}{kg}}\times TiO_{2}\;feed}{TiO_{2}\;feces}-8.22\\ \times \left(N\;feed-\frac{N\;feces\times TiO_{2}\;feed}{TiO_{2}\;feces}\right))\; \end{array}$$ (5)

#### 2.3.2 Statistical analysis

Data were analyzed using IBM SPSS Statistics v30.0. An initial planned comparison between STD and REF was conducted using a *t*-test to verify differences caused by reformulation. Subsequently, a one-way ANOVA including STD, REF and APP was applied as the final statistical analysis. When significant, multiple comparisons among means were performed using Tukey's *post-hoc* test. Differences were considered significant with *P* ≤ 0.05, while tendencies with *P* ≤ 0.10.

## 3 Results

### 3.1 Meta-analysis

The analyzed database included 35 trials, with a duration of 34–48 days (avg. 40 days) and different mBOA inclusion levels: 200 mg/kg (seven trials), 250 mg/kg (three trials), 300 mg/kg (17 trials), and 500 mg/kg (eight trials).

The pooled effects of mBOA supplementation on broiler growth performance are reported in Table 3. ADFI increased in 54.3% of the trials with an average increase of 0.128 g/day in mBOA group compared with control (*P* = 0.854; τ^2^ = 16.17). Supplementation with mBOA consistently improved ADG and FBW in 82.8% and 88.6% of the evaluated trials, with an average increase of 0.988 g/day (*P* = 0.017; τ^2^ = 4.79; Figure 2) and 38.3 g (*P* = 0.008; τ^2^ = 6,465.14; Figure 3), respectively. FCRst was improved in 82.8% of the trials following mBOA supplementation, with an average reduction of four points (*P* = 0.001; τ^2^ = 0.003; Figure 4). Mortality data were available for 31 studies, with a reduction compared to the control group observed in 56.3% of them, and a non-significant average difference of −0.11% (*P* = 0.613; τ^2^ = 1.42).

**Table 3:** Effect of mBOA supplementation on whole life growth performance in broiler chickens across trials included in the meta-analysis.

Item	Control	mBOA	P-value	Confidence interval	95% prediction interval
ADFI (g)	104.82 ± 4.74	105.02 ± 4.87	0.854	−1.28 to 1.53	−8.17 to 8.43
ADG (g)	60.20 ± 2.55	61.29 ± 2.78^*^	**0.017**	0.19 to 1.79	−3.54 to 5.51
FBW (g)	2,466.08 ± 78.25	2,504.16 ± 74.89^*^	**0.008**	10.53 to 66.12	−127.61 to 204.26
FCRst^a^	1.74 ± 0.04	1.70 ± 0.04^*^	**0.001**	−0.06 to −0.02	−0.15 to 0.08
Mortality^*b*^ (%)	3.92 ± 1.93	3.82 ± 2.01	0.613	−0.55 to 0.33	−2.59 to 2.37

Values are expressed as arithmetic mean ± SD across studies and provided for descriptive purposes. *P*-values refer to the pooled mean difference obtained from the meta-analysis (random-effects, REML, truncated Knapp–Hartung) and asterisks within a row indicate significant differences (*P* ≤ 0.05). The 95% confidence interval (CI) reflects the precision of the pooled mean effect, while the 95% prediction interval (PI) reflects the expected range of the true effect in a new trial, given the between-study heterogeneity observed.

ADFI, average daily feed intake; ADG, average daily gain; FBW, final body weight; FCRst, standardized FCR.

^a^FCR standardized (−0.01 FCR for each extra 50 g of FBW). ^b^Mortality data were available for 31 studies.

**Figure 2:**
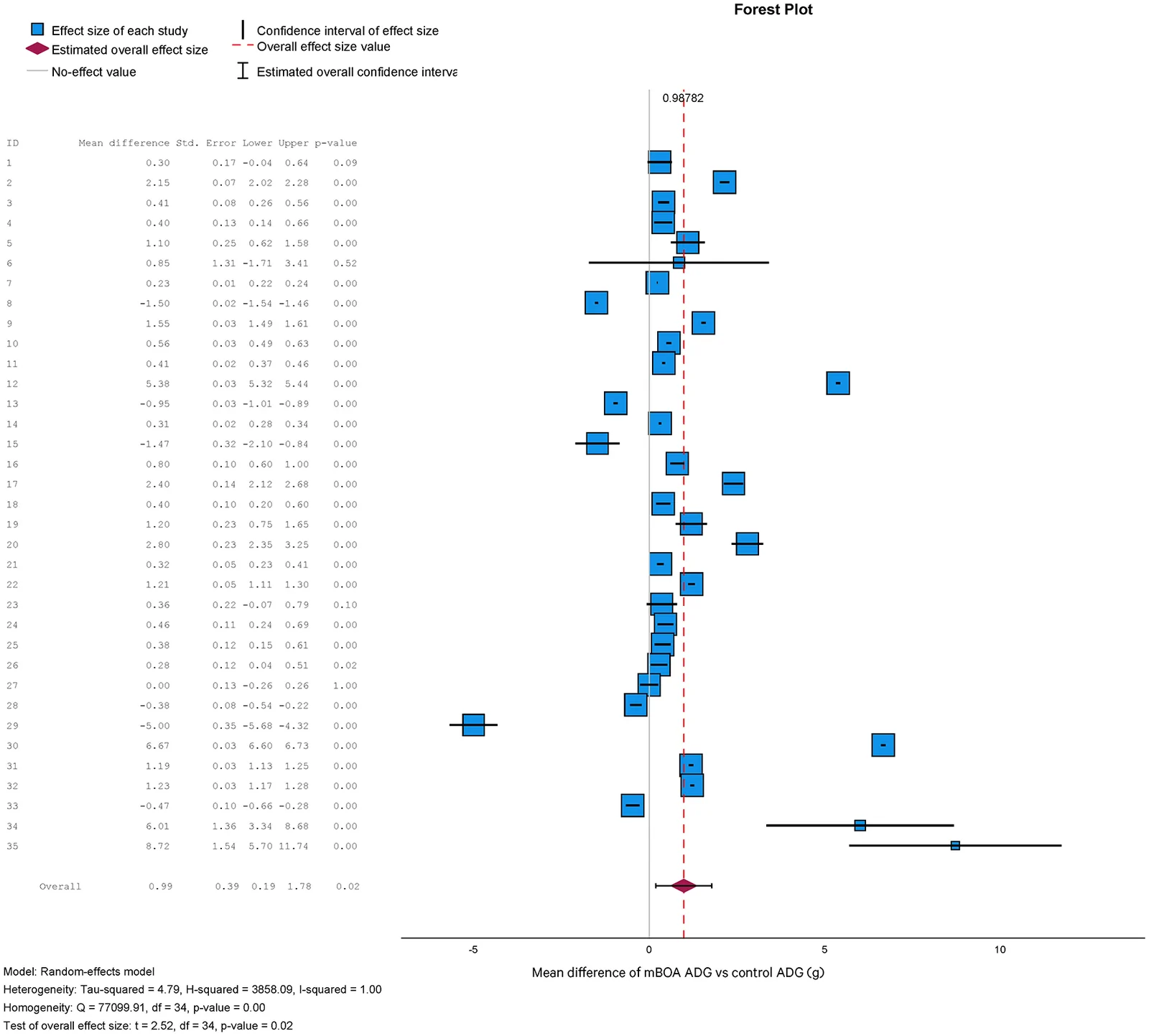
Forest plot of the effect of mBOA supplementation on average daily gain (ADG) across trials included in the meta-analysis.

**Figure 3:**
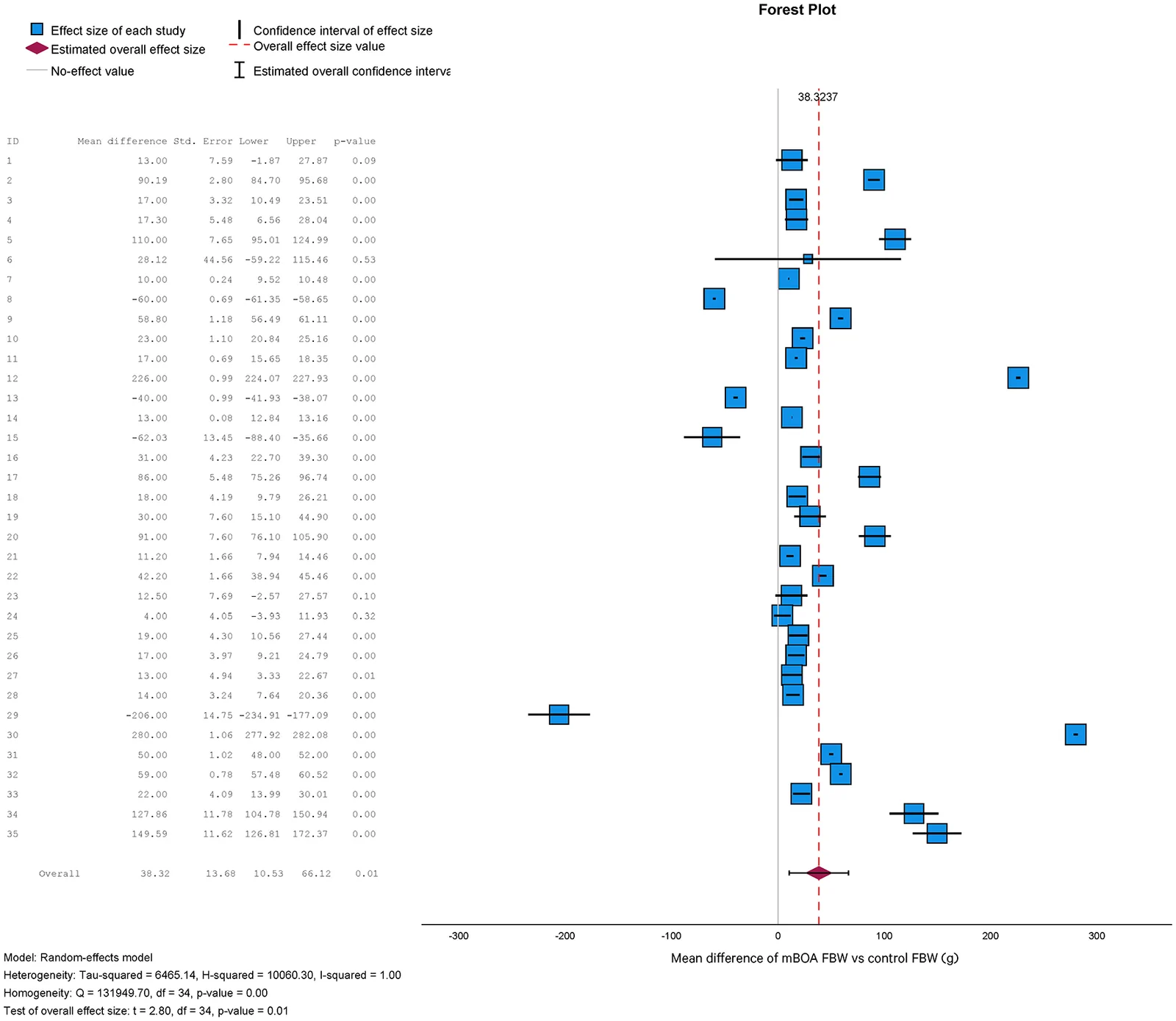
Forest plot of the effect of mBOA supplementation on final body weight (FBW) across trials included in the meta-analysis.

**Figure 4:**
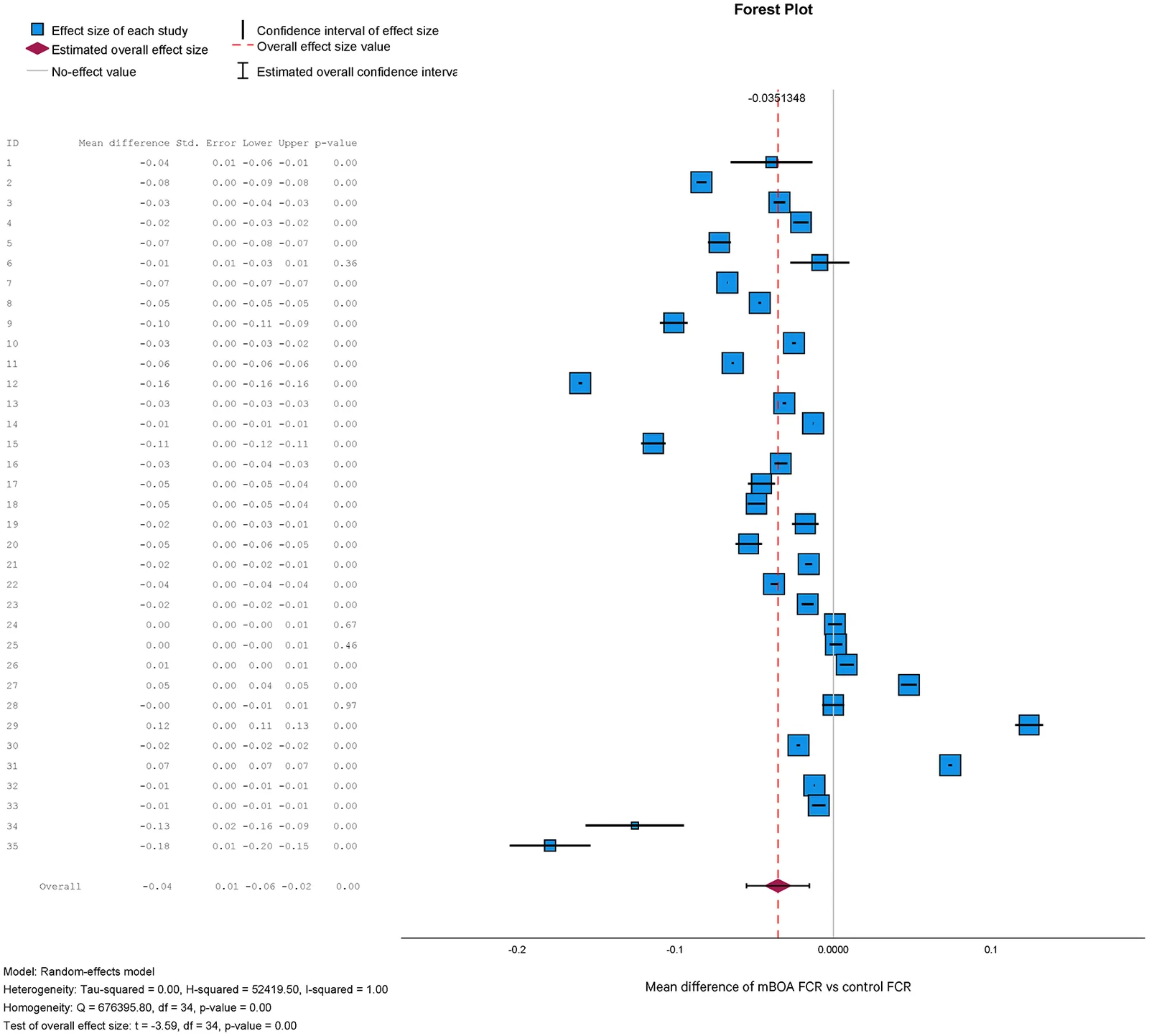
Forest plot of the effect of mBOA supplementation on standardized FCR (FCRst) across trials included in the meta-analysis.

Substantial between-study heterogeneity was observed across outcomes and is summarized by τ^2^ in the forest plots of the observed statistically significant parameters. Funnel plots and Egger's regression-based tests were performed for all outcomes. No significant funnel asymmetry was detected for ADG (intercept = 0.48, *P* = 0.266; Supplementary Figure S1), FCRst (intercept = −0.019, *P* = 0.128; Supplementary Figure S2), ADFI (intercept = −0.049, *P* = 0.951), or mortality (intercept = −0.013, *P* = 0.967); a significant asymmetry was detected for FBW (intercept = 46.29, *P* = 0.011; Supplementary Figure S3). When mBOA dose was included as a covariate in the same analysis, the residual funnel asymmetry for FBW was no longer significant (intercept = 25.24, *P* = 0.582).

A subgroup analysis by trial type (23 experimental vs. 12 field) showed consistent effect direction across both subgroups for all outcomes, with a numerically larger effect in field trials for FBW (+29.95 g experimental vs. +54.24 g field), ADG (+0.84 vs. +1.23 g/day), FCRst (−0.033 vs. −0.039), and ADFI (−0.22 vs. +0.67 g/day). The test of subgroup homogeneity was non-significant for all four outcomes (FBW: *P* = 0.452; ADG: *P* = 0.643; FCRst: *P* = 0.774; ADFI: *P* = 0.600), indicating no statistically significant difference between experimental and field trial estimates. A dose meta-regression (200–500 mg/kg) did not detect a significant dose-response slope for any outcome: FBW (*b* = 0.072, *P* = 0.619), ADG (*b* < 0.001, *P* = 0.907), FCRst (*b* < −0.001, *P* = 0.606), ADFI (*b* = −0.002, *P* = 0.772).

A leave-one-out sensitivity analysis for FBW (35 iterations) showed the pooled estimate ranged from +31.18 g to +45.38 g and remained statistically significant (*P* ≤ 0.016) in all iterations, confirming that no single trial drove the overall result.

### 3.2 Performance-derived nutritional value

Using the FCRst improvement outcomes from the meta-analysis, the performance benefit was translated into a nutritional value for mBOA at 300 mg/kg. Applying the calculation framework detailed in the Materials and Methods section, the observed reduction was back-converted into diet-level energy and protein recovered and then normalized to the additive inclusion to obtain ME[mBOA] and CP[mBOA].

At the target inclusion (300 mg/kg), the nutritional value of mBOA corresponded to 60.6 kcal ME/kg feed and 3.89 g CP/kg feed, with digestible amino acids allocated as a fixed proportion. When accounting for the 95% *CI* related to FCRst, the CI range for ME and CP correspond to 26.0–95.2 kcal ME/kg feed and 1.7–6.1 g CP/kg feed, respectively.

#### *3.3 In vivo* validation trial

#### 3.3.1 Proximate analysis of experimental diets

The basal feed used for the REF and APP groups was identical, and for this reason only samples of the standard and reformulated diets were submitted for chemical analysis. In addition, finisher diets with and without TiO_2_ were produced from the same basal diet, therefore only Finisher II sample without TiO_2_ was analyzed. Results of proximate analysis are reported in Table 4. Diet reformulation induced a reduction in CP content (3–6 g/kg; −2.2% on average) and crude fat (5–15 g/kg; −25.7% on average), whereas crude fiber showed only minor changes (−2 to −1 g/kg; +5.8% on average). Crude ash content was almost unaffected, while gross energy was reduced by 31–57 kcal/kg of diet, corresponding to an average of −1.1%.

**Table 4:** Proximate analysis of standard and reformulated diets fed in the validation trial.

	Starter			Grower			Finisher		
Item	Standard diet	Reformulated diet	Delta	Standard diet	Reformulated diet	Delta	Standard diet	Reformulated diet	Delta
Moisture^a^ (g/kg)	120	120	0	117	117	0	116	116	0
Crude protein^b^ (g/kg)	215	211	−4	198	192	−6	182	179	−3
Crude fat^c^ (g/kg)	45	40	−5	45	36	−9	53	38	−15
Crude fiber (g/kg)	27	29	+2	27	29	+2	27	28	+1
Crude ash^d^ (g/kg)	51	51	0	45	45	0	41	40	−1
Gross heat^e^ (kcal/kg)	4,039	4,008	−31	4,070	4,013	−57	4,073	4,025	−48

Methods: ^a^at 103 °C for 4 h; ^b^Kjeldahl (*N* = 6.25); ^c^acid hydrolyzation; ^d^determined at 550 °C; ^e^bomb calorimeter.

#### 3.3.2 Growth performance

The growth performance results are reported in Table 5.

**Table 5:** Growth performance results in broiler chickens during the *in vivo* validation trial.

Item	STD	REF	APP	SEM	*P*-value (STD vs. REF)^1^	*P*-value (all groups)^2^
Starter (0–14d)						
d14–BW (g)	502	501	500	2	0.90	0.97
ADFI (g/day)	38.1	38.8	38.0	0.2	0.09	0.10
ADG (g/day)	32.8	32.9	32.7	0.2	0.94	0.99
FCR	1.16a	1.18^*^b	1.16a	0.01	**0.004**	**0.006**
Grower (14–28d)						
d28–BW (kg)	1.73	1.72	1.72	0.01	0.60	0.77
ADFI (g/day)	125.2	127.2	126.0	0.5	0.14	0.27
ADG (g/day)	87.7	87.0	86.5	0.5	0.62	0.71
FCR	1.43	1.46^*^	1.46	0.01	**0.03**	0.09
Finisher (28–41d)						
d41–BW (kg)	3.26	3.25	3.25	0.02	0.85	0.98
ADFI (g/day)	201.8	206.0	205.7	1.1	0.13	0.21
ADG (g/day)	117.5	117.8	118.9	0.9	0.90	0.81
FCR	1.72	1.75	1.73	0.01	0.23	0.36
Overall (0–41d)						
ADFI (g/d)	119.6	121.8	120.7	0.6	0.07	0.17
ADG (g/d)	78.2	78.1	78.3	0.4	0.88	0.97
FCR	1.53a	1.56^*^b	1.54ab	0.01	**0.003**	**0.003**

^1^*P*-value refers to an initial planned comparison between STD and REF, conducted using a *t*-test to verify differences caused by reformulation.

^2^*P*-value refers to one-way ANOVA, including STD, REF and APP.

Asterisks within a row indicate a significant difference (*P* ≤ 0.05) between STD and REF following *T*-test.

Values within a row with different letters differ significantly (*P* ≤ 0.05) according to one-way ANOVA followed by Tukey's post-hoc test.

The primary scope of the study was to create a performance gap between STD and REF groups, by reducing the dietary nutrient density. The objective was accomplished since, to achieve a comparable FBW to STD over the whole period, birds in REF group consumed more feed (121.8 g/day vs. 119.6 g/day; *P* = 0.07) and consequently exhibited a higher and statistically different FCR (1.56 vs. 1.53; *P* = 0.003).

Considering all the groups, no significant differences in body weight were detected at the end of any experimental phase. In the starter phase (d 0–14), REF showed a significant loss in feed efficiency compared to STD, while APP restored FCR to STD levels (*P* = 0.006). During the grower phase (d 14–28), a trend in favor of STD compared to REF and APP was observed for FCR. In the finisher phase (d 28–41), FCR was numerically higher for REF (1.75) compared to STD and APP (1.72 and 1.73, respectively). Considering the overall period (d 0–41), REF had a numerically higher ADFI (121.8 g) compared to STD (119.6 g) and APP (120.7 g; *P* = 0.174). These differences led to significant changes in overall FCR, which was higher in REF group compared to STD (1.56 vs. 1.53; *P* = 0.003), with APP achieving an intermediate value (1.54), not statistically different from either group, but numerically closer to STD.

#### 3.3.3 Nutrient digestibility

Table 6 summarizes nutrient digestibility results.

**Table 6:** Nutrient digestibility results measured from pooled fecal samples collected at day 31–32 of the *in vivo* validation trial.

Item	STD	REF	APP	SEM	*P*-value (STD vs. REF)	*P*-value (all groups)
Dry matter (%)	89.37	90.17^*^	89.52	0.24	**0.02**	0.07
Crude protein (%)	59.56a	60.43ab	61.55b	0.50	0.28	**0.04**
Crude fat (%)	80.58a	76.94^*^b	78.87a	0.46	**< 0.001**	**< 0.001**
Crude ash (%)	30.90ab	29.35b	31.69a	0.53	0.08	**0.01**
Gross energy (%)	70.79	71.18	71.52	0.21	0.23	0.08
AMEn (kcal/kg DM)	2,873a	2,827^*^b	2,852ab	9	**< 0.001**	**0.003**

Asterisks within a row indicate a significant difference (*P* ≤ 0.05) between STD and REF following *T*-test. Values within a row with different letters differ significantly (*P* ≤ 0.05) according to one-way ANOVA followed by Tukey's post-hoc test.

When comparing STD and REF, reformulation significantly increased DM digestibility (*P* = 0.02), reduced crude fat digestibility (*P* < 0.001), and tended to decrease digestibility of crude ash (*P* = 0.08). While no differences were registered for gross energy, a significant reduction in AMEn digestibility (*P* < 0.001) was measured for REF (2,827 kcal/kg DM) compared to STD (2,873 kcal/kg DM).

Considering all groups, APP showed higher CP digestibility compared to both STD and REF (*P* = 0.04). Moreover, crude fat digestibility was improved in APP compared to REF, reaching values comparable to STD (*P* < 0.001). APP tended to increase gross energy digestibility (*P* = 0.08) and displayed an intermediate response for AMEn digestibility (2,852 kcal/kg DM; *P* = 0.003).

## 4 Discussion

Within a cost-constrained production landscape, this study demonstrates that the use of a scientifically-validated gut-health feed additive can be translated into diet formulation value. A large-scale meta-analysis quantified consistent gains in growth efficiency thanks to the supplementation of mBOA. The meta-analysis outcomes were parameterized into a performance-derived nutritional value, that was finally successfully evaluated *in vivo* using a reduced-density diet design.

Starting from the rationale that intestinal homeostasis is fundamental for high-level production in poultry industry, the hypothesis was that the mBOA demonstrated multifactorial mechanism of action could have a role in improving dietary nutrient utilization. mBOA reinforces intestinal barrier, also during inflammatory challenges, by increasing the expression of tight junctions and protecting epithelial integrity, thus contributing to the maintenance of a better gut functionality (13, 16). Moreover, mBOA favors a better intestinal structure and morphology (15, 17), expanding the nutrient absorption surface. mBOA also promotes a favorable modulation of the intestinal microflora (14). A further mechanism of action implied in feed efficiency is the role of mBOA in improving nutrient digestion, with enhanced activity of endogenous epithelial cell membrane-bound enzymes, other than increased expression and function of nutrient transporters (15). In this study, mBOA increased the digestibility of nutrients and energy of a reformulated diet, confirming prior evidences and further demonstrating the nutritional role of the feed additive.

Previous literature indicates that immune activation represents a metabolic cost by diverting nutrients away from productive functions toward the support of inflammatory responses (10–12). Inflammation also impairs insulin sensitivity and promotes lipid mobilization to sustain immune activation (18). The control of inflammatory processes is therefore expected to improve insulin homeostasis and reduce circulating lipid flux toward peripheral tissues. In this context, mBOA supplementation appears to modulate the inflammation-insulin axis, as reflected by improvements in meat lipid profile and oxidative stability already demonstrated in previous studies (19). While a direct re-assessment of all these mechanisms was outside the scope of the present study, this existing body of evidence provided the framework on which the meta-analysis and performance-derived nutritional value approach was built.

The translation of the above-mentioned mechanisms into animal benefit was demonstrated by the robust meta-analytic evidence of performance improvements, despite the substantial heterogeneity related to the working database that combined small experimental trials and large-scale field trials across diverse genetics, countries, and mBOA dose levels (200–500 mg/kg). The supplementation of the microencapsulated blend of botanicals and organic acids did not show significant changes in ADFI, but rather exerted a statistical reduction of four FCRst points, corresponding to a saving of approximately 40 g of feed per kg of body weight gain. This suggests that the improvement in feed efficiency was effectively driven by an enhancement of nutrient utilization, rather than intake reduction. A significant effect on ADG and FBW was also observed at the meta-analysis level. Subgroup analysis, dose meta-regression, funnel plots, and a leave-one-out sensitivity analysis all supported the consistency of the pooled effects across trial types and individual studies, allowing a more balanced interpretation of the results in the context of the heterogeneous nature of the historical database. A significant funnel plot asymmetry was detected only for FBW, likely a consequence of the already discussed database heterogeneity, also considering that no trial was excluded from the meta-analysis based on the nature of its outcome. This significance disappeared when mBOA dosage was considered as a covariate in the analysis, indicating that it primarily reflected the uneven distribution of dose levels across trials, with higher prevalence of 300 mg/kg.

The observed improvement on productive parameters can be related to the previously discussed effects of mBOA on gut-health homeostasis, lower cost of inflammation, and better utilization of nutrients, suggesting a consistent response across genetics, regions, and production settings. Finally, no differences were observed in overall mortality between mBOA and control groups, consistently with the inclusion of trials conducted under non-challenged conditions and with the intended use of the zootechnical additive in healthy animals. From a formulation perspective, growth responses were considered supportive outcomes, while FCRst was selected as the most appropriate parameter to translate performance improvements into nutritional values.

The practical quantification of the nutritional value of mBOA at 300 mg/kg was calculated using the meta-analysis performance outcomes. The formulation value (60.6 kcal ME/kg feed; 3.9 g CP/kg feed), while representing a performance-derived estimate from the meta-analysis outcomes, helps narrow the gaps left open among mechanism of action, productive benefits, and practical application in animal nutrition. This approach can be considered a novel methodology for correlating meta-analysis evidence with nutritional values parameterization.

The final step of this research was the proof-of-use of mBOA performance-derived nutritional value during an *in vivo* trial in broilers under reduced-density diets. The proximal analysis confirmed the theoretical 2% reduction of CP content in the reformulated diet compared to the standard feed. Although the reformulation aimed at a theoretical reduction of 80 kcal/kg of dietary energy, proximate analysis revealed an average decrease of approximately 45 kcal/kg across feeding phases. This discrepancy may be explained by the actual energy values of the raw materials used to produce diets for the *in vivo* trial, that may not fully reflect the theoretical values of the reformulation scheme. Moreover, as the reformulation primarily targeted soybean oil inclusion, a marked reduction in crude fat was observed with proximate analysis, that could likely indirectly contribute to the loss of gross and metabolizable energy of the reformulated diets (20).

The reformulation achieved the intended performance outcome, resulting in decreased growth performance (−3 points FCR in the overall period) and digestibility of nutrients (−46 kcal AMEn/kg DM) in REF animals compared to the STD group. To reach the same body weight, REF animals compensated by increasing feed intake, which consequently impaired feed efficiency. This physiological response is driven by the attempt of the bird to adjust feed consumption to satisfy the energy requirement and it is strictly related to the dietary energy density (21, 22). Consistently, a recent research demonstrated that the energetic density of the diet regulates feed intake in broilers through the gut-brain axis, involving appetite-related hormones, hypothalamic signaling, and gut health status (23). The APP group mitigated the loss of performance caused by the reformulation, recovering feed intake values and consequently yielding to an overall FCR that was intermediate and not statistically different from either STD or REF, while numerically close to the STD group. These results are also supported by the observed improvement of nutrient digestibility and energy utilization in animals supplemented with mBOA, which were comparable to or higher than those of the STD group. This improvement numerically corresponded to a recovery of 25 kcal out of the 46-kcal reformulation gap, with APP AMEn value statistically intermediate between REF and STD, supporting the overall rationale that mBOA contributes to recover nutrient utilization losses caused by diet reformulation. Protein was the second parameter targeted by the reformulation and evaluated in the digestibility trial. As widely reported, dietary protein availability plays a key role in determining body weight gain and feed efficiency (24). In the present study, the significant improvement in CP digestibility observed in APP compared with STD reached almost 2% (3.77 g CP/kg feed relative to a 19% CP diet), corresponding to the nutritional value of 3.88 g CP/kg feed. These findings support the contribution of mBOA to enhanced protein utilization, as already reported by Toschi et al. (15), which likely plays a relevant role in sustaining performance.

Consistent with the nutritional value approach described above, the *in vivo* trial confirmed its relevance specifically for reduced-density diet formulations, where mBOA supplementation helped recover performance and nutrient digestibility losses associated with the reformulation. A major strength of the present work is the integrated multi-step approach, which involved a validation of the mechanisms of action reported for mBOA and their subsequent translation into practical nutritional value. The main limitation was the conduction of the *in vivo* validation in a single research facility, that could only partially resemble the real field conditions. To mitigate this constraint, a high bird density was adopted (approximately 14 birds/m^2^) in order to mimic the typical environmental stress from commercial settings. Further *in vivo* confirmation studies will be needed to validate the performance-derived nutritional value under different scenarios, including different feed formulations and densities, mBOA dose-responses or environmental challenges. Such studies will also allow further refinement of the precision of the proposed mBOA values for ME and CP, across broader range of conditions. Additional noteworthy perspectives to be investigated will likely be the economic impact of mBOA use under reformulation and a sustainability study to understand the impact of mBOA potential reduction in environmental footprint by lowering the feed use per kg of body weight gain.

## 5 Conclusion

This study demonstrates that the effects of a gut health feed additive for broilers can be translated from scientifically validated qualitative biological benefits into quantitative nutritional values that can be used during feed formulation. Through a large scale 35-trial meta-analysis, a performance-derived nutritional set of values was established for a microencapsulated blend of botanicals and organic acids (mBOA) feed additive, to be used particularly in reduced-density reformulation scenarios. This was finally evaluated *in vivo* in broiler chickens receiving reduced-density diets.

The results show that this approach allows recovery of energy and protein gaps introduced by diet reformulation, preserving broiler feed efficiency without compromising growth performance. Collectively, these findings support a formulation-oriented perspective of gut health additives, where improvements in intestinal functionality and health can become reliable nutritional values, allowing greater flexibility, cost control, and sustainability in broiler diet formulation.

## Data Availability

The raw data supporting the conclusions of this article will be made available by the authors, without undue reservation.
