# Supplementary material for: Development of a performance-derived nutritional value for a microencapsulated gut health additive from a meta-analysis of 35 trials and its in vivo validation in broilers
Source: Front Vet Sci. 2026 Oct 9;13:1888365. doi: 10.3389/fvets.2026.1888365 (PMC13692780; doi:10.3389/fvets.2026.1888365)

1 **Figure S1. Funnel plot of mean difference against standard error for average daily gain (ADG), assessing asymmetry across trials included in the meta-analysis.**

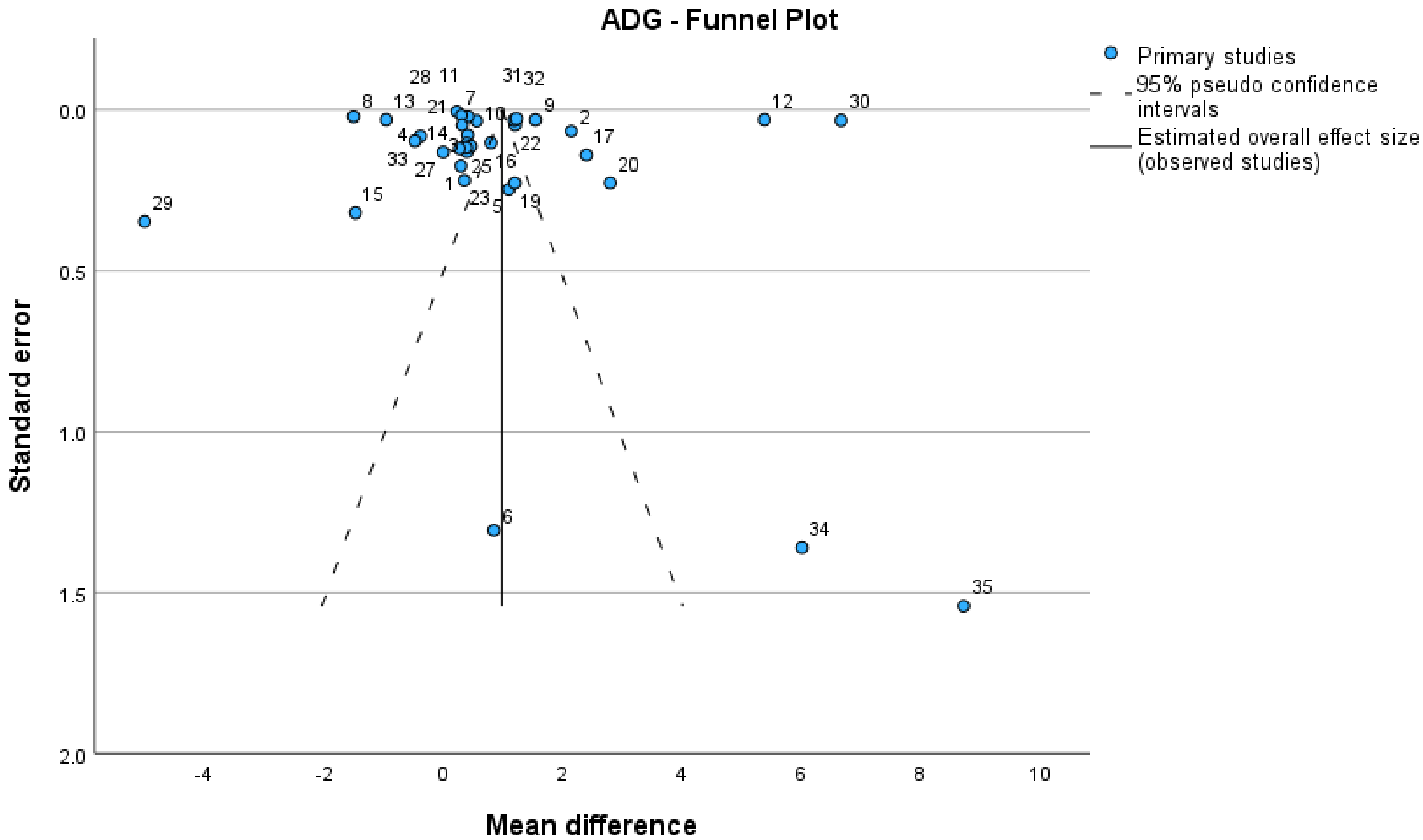

2 **Figure S2. Funnel plot of mean difference against standard error for standardized FCR (FCRst), assessing asymmetry across trials included in the meta-analysis.**

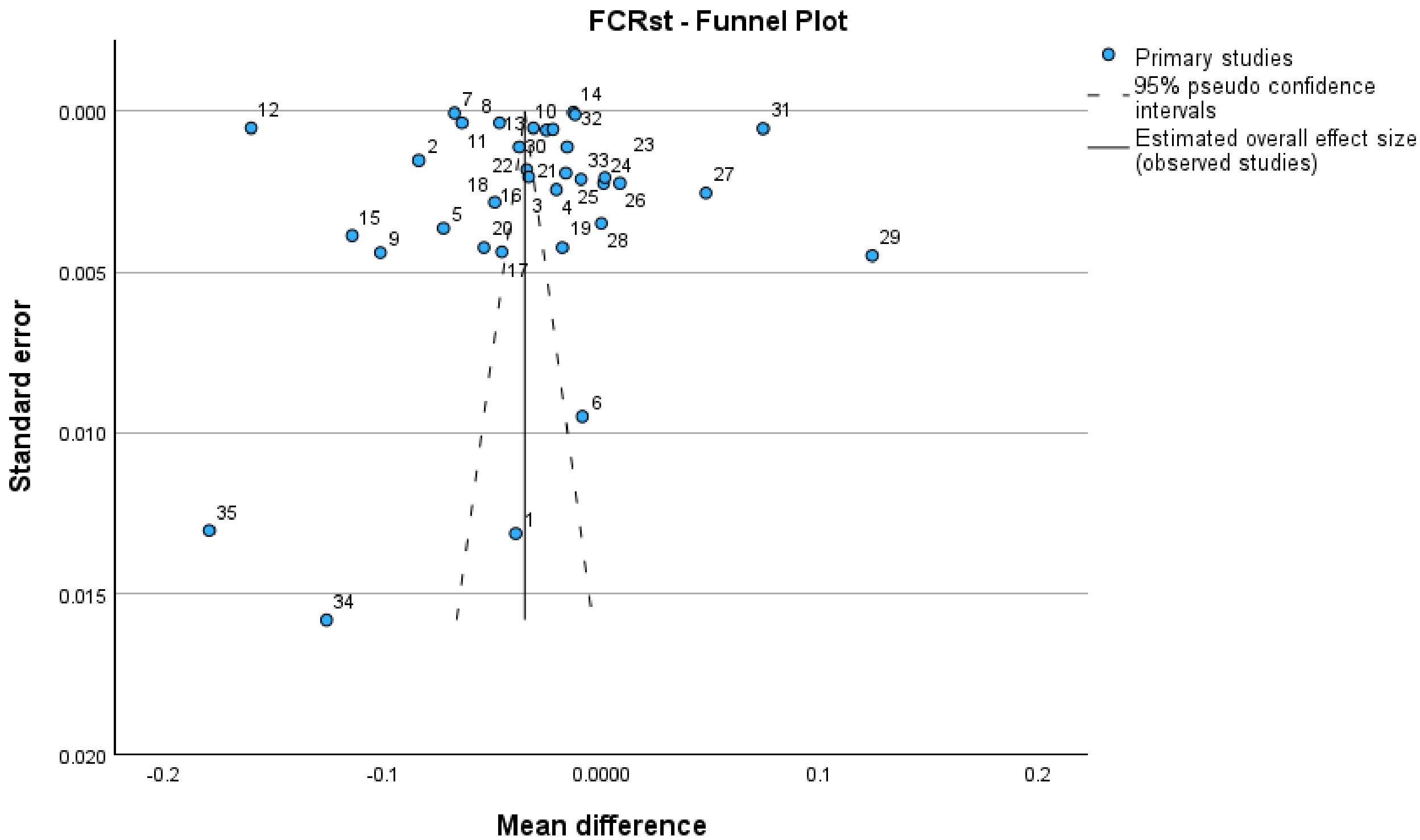

3 **Figure S3. Funnel plot of mean difference against standard error for final body weight (FBW), assessing asymmetry across trials included in the meta-analysis.**

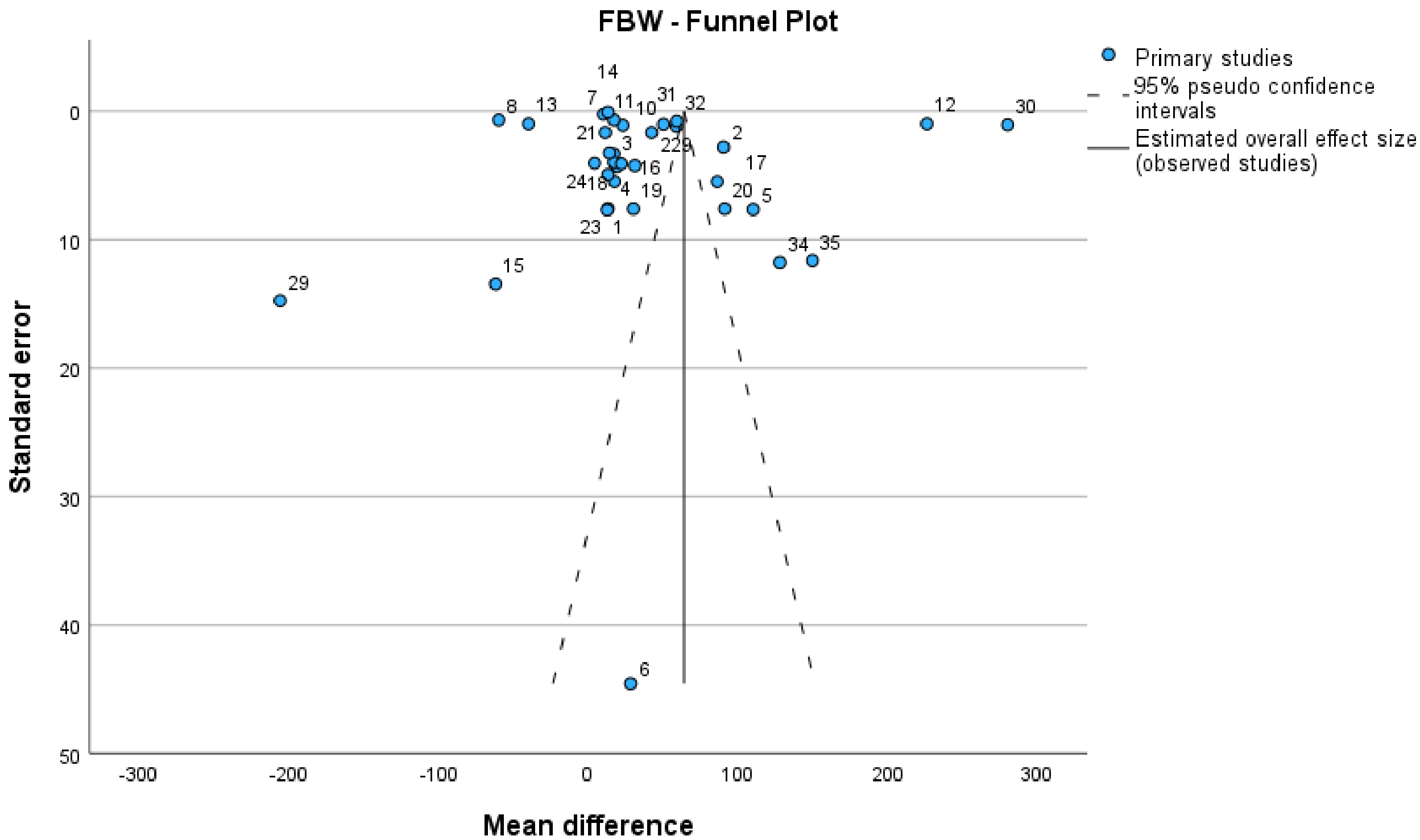

Supplement: Supplementary file 1 [file Data_Sheet_1.pdf]
